# Antioxidant and Anti-Inflammatory Profiles of Spent Coffee Ground Extracts for the Treatment of Neurodegeneration

**DOI:** 10.1155/2021/6620913

**Published:** 2021-05-19

**Authors:** Simone Angeloni, Michela Freschi, Pasquale Marrazzo, Silvana Hrelia, Daniela Beghelli, Ana Juan-García, Cristina Juan, Giovanni Caprioli, Gianni Sagratini, Cristina Angeloni

**Affiliations:** ^1^School of Pharmacy, University of Camerino, Via Sant' Agostino 1, I-62032 Camerino (MC), Italy; ^2^International Hub for Coffee Research and Innovation, I-62020 Belforte del Chienti (MC), Italy; ^3^Department for Life Quality Studies, Alma Mater Studiorum University of Bologna, Rimini 47921, Italy; ^4^School of Biosciences and Veterinary Medicine, University of Camerino, Via Gentile III da Varano, 62032 Camerino (MC), Italy; ^5^Laboratory of Food Chemistry and Toxicology, Faculty of Pharmacy, University of Valencia, Av. Vicent Andrés Estellés s/n, 46100 Burjassot, València, Spain

## Abstract

Spent coffee grounds (SCGs), waste products of coffee beverage production, are rich in organic compounds such as phenols. Different studies have demonstrated phenol beneficial effects in counteracting neurodegenerative diseases. These diseases are associated with oxidative stress and neuroinflammation, which initiates the degeneration of neurons by overactivating microglia. Unfortunately, to date, there are no pharmacological therapies to treat these pathologies. The aim of this study was to evaluate the phenolic content of 4 different SCG extracts and their ability to counteract oxidative stress and neuroinflammation. Caffeine and 5-*O*-caffeoylquinic acid were the most abundant compounds in all extracts, followed by 3-*O*-caffeoylquinic acid and 3,5-*O*-dicaffeoylquinic acid. The four extracts demonstrated a different ability to counteract oxidative stress and neuroinflammation in vitro. In particular, the methanol extract was the most effective in protecting neuron-like SH-SY5Y cells against H_2_O_2_-induced oxidative stress by upregulating endogenous antioxidant enzymes such as thioredoxin reductase, heme oxygenase 1, NADPH quinone oxidoreductase, and glutathione reductase. The water extract was the most effective in counteracting lipopolysaccharide-induced neuroinflammation in microglial BV-2 cells by strongly reducing the expression of proinflammatory mediators through the modulation of the TLR4/NF-*κ*B pathway. On these bases, SCG extracts could represent valuable nutraceutical sources for the treatment of neurodegeneration.

## 1. Introduction

The food industry generates considerable amounts of waste products that require to be appropriately managed to reduce their negative sustainability impacts. An appropriate waste management helps to reduce not only the negative effects on the environment but also has got an important economic impact, since there is less production of nonrenewable resources and less energy is used in the production of new goods. Among food industry wastes, coffee by-products have been extensively taken into consideration for recycle [1–5]. Coffee is made by roasting and grinding coffee beans to produce a powder that is extracted with hot water or brewed. During the preparation of coffee beverages, a solid residue known as spent coffee grounds (SCG) is produced and this is the most abundant coffee waste (55−67%) [6].

About 650 kg of SCG are produced from 1000 kg of green coffee beans, and nearly 2 kg of wet SCG are obtained by the preparation of 1 kg of soluble coffee [7]. SCG is a nonedible resource, which is not entering into the food chain, and its disposal in the environment is dangerous since SCG contains caffeine, tannins, and polyphenols that make it a toxic residue [6, 7]. On these bases, numerous authors have suggested different ways to recycle SCG, to manage and reduce its disposal [8–10]. SCG can be used as a source of oil for biodiesel production [11–13] or as a source of recoverable sugars which can be employed as food addictive or for bioethanol production [13–16]. Moreover, different papers focused on SCG constituents and their application in the food and nutraceutical industry [1, 17–19]. The main constituents of SCG are polysaccharides, proteins, and lipids, as well as minerals, caffeine, melanoidins, and phenols [20]. Phenols of SCG are mainly represented by different highly bioavailable and bioactive phenolic acids such as chlorogenic, caffeic, ellagic, *trans*-ferulic, gallic, p-hydroxybenzoic, p-coumaric, protocatechuic and tannic acids, and flavonoids such as catechin, epicatechin, rutin, and quercetin [1, 21, 22]. Phenolic compounds are well known for their beneficial effects on human health, e.g., in the prevention of different chronic degenerative diseases such as cancer, cardiovascular, and neurodegenerative diseases [23–25]. Neurodegenerative diseases, mainly including Parkinson's and Alzheimer's diseases, are a health problem primarily affecting the elderly. These disorders share common cellular and molecular events such as oxidative stress, abnormal protein deposition, damaged mitochondrial function, induction of apoptosis, impairment of proteostasis, and neuroinflammation [26]. Neuron cells are particularly vulnerable to oxidative damage due to their high polyunsaturated fatty acid content in membranes, high oxygen consumption, and weak antioxidant defenses [27]. Oxidative damage results in an increase in reactive oxygen species (ROS), which leads to further oxidative damage and feeds this self-propagating cycle. ROS may also trigger protein misfolding, potentially leading to protein aggregation, which is a classical hallmark of neurodegenerative diseases such as Alzheimer's and Parkinson's diseases [28].

In addition to oxidative damage, in recent years, the immune system is emerging as a key determinant in the onset and progression of neurodegeneration [29, 30] as it triggers modification of cytokine signaling, immune cell proliferation and migration, impaired phagocytosis, and reactive gliosis [31]. Neuroinflammation, caused by the activation into proinflammatory states of the brain immune cells, namely, microglia and astrocytes, represents a fundamental defense system that protects neurons from toxic substance and microorganisms. In normal physiological conditions, this is commonly a positive mechanism aimed at preserving the brain integrity by removing threats and reestablishing homeostasis [32]. However, chronic neuroinflammation can stimulate a series of events that induce progressive neuronal damage that characterizes many neurodegenerative disorders [33]. Unfortunately, currently, no drugs capable of slowing down or blocking the progression of these debilitating pathologies have been identified. This is why the research is turning its attention to the identification of natural compounds with a preventive/protective activity against neurodegenerative disorders. As we previously demonstrated that extracts obtained by coffee silverskin, another coffee by-product, are rich in bioactive compounds with antioxidant and antibacterial activities, we assumed that also SCG could be rich in bioactive phytochemicals with potential neuroprotective activity [5, 34].

The present study was undertaken to evaluate the phenolic content of 4 different SCG extracts and their ability to counteract oxidative stress and neuroinflammation in neuron-like SH-SY5Y and microglial BV-2 cells.

## 2. Materials and Methods

### 2.1. Chemicals and Reagents

Cyanidin 3-glucoside chloride, delphinidin 3,5-diglucoside chloride, and kaempferol 3-glucoside were purchased from PhytoLab (Vestenbergsgreuth, Germany). The other 27 analytical standards of the 30 bioactive compounds and high-glucose Dulbecco's modified Eagle medium (DMEM), fetal bovine serum (FBS), penicillin, streptomycin, glutamine, LPS from Escherichia coli serotype O127: B8, 3-(4,5-dimethylthiazol-2-yl)-2,5-diphenyl tetrazolium bromide (MTT), 2,7-dichlorodihydrofluorescein diacetate (DCFH-DA), H_2_O_2_, dimethyl sulfoxide (DMSO), were purchased from Sigma Aldrich–Merck (Milan, Italy). The 30 analytical standards were dissolved in pure standard compounds in HPLC-grade methanol at a concentration of 1000 mg L^−1^ and stored in glass stoppered bottles at 4°C. The standard working solutions were obtained by appropriate dilution of the stock solution with methanol. HPLC-grade formic acid 99–100% was purchased from Merck (Darmstadt, Germany) while HPLC-grade methanol (MeOH) and ethanol (EtOH) were supplied by Carlo Erba (Milano, Italy). Deionized water was obtained from a Milli-Q Reagent Water System (Bedford, MA, USA). All other solvents and chemicals were of analytical grade. Before HPLC analysis, all samples were filtered with Phenex RC 4 mm 0.2 *μ*m syringeless filter, Phenomenex (Castel Maggiore, Italy). Low-endotoxin FBS was purchased from Euroclone (Milan, Italy).

### 2.2. Spent Coffee Ground Sample and Extract Preparation

Roasted beans of 100% Coffea arabica L., Ethiopian origin, were supplied by Simonelli Group S.p.A. (Belforte del Chienti, Italy). Roasted beans were grinded by Mythos 1 grinder (Simonelli Group S.p.A.), and spent coffee ground (SCG) was obtained after a series of replicates of espresso coffee preparations using a VA833 Black Eagle espresso coffee machine (Victoria Arduino, Simonelli Group S.p.A., Belforte del Chienti, Italy). The extraction of espresso coffee was carried out as follows: 7 ± 0.05 g of roasted and ground (R&G) coffee per cup, 25 ± 1 s of extraction, water pressure and temperature 9 bar and 92.0°C, respectively, and 25 ± 2 g in cup. SCG samples were collected and oven-dried at 50°C until constant weight (about 48 h). Dried SCG sample was stored at 4°C up to use. The extract preparation was carried out following a previous work [5] with some adjustments. For the current research, four extracts were selected on the base of their high performance in terms of bioactive compound recovery and extraction yield [5, 21]. Briefly, 10 g of SCG were extracted with 50 mL of solvent assisted by a FALC ultrasonic bath (FALC, Treviglio, Italy) at a frequency of 40 kHz for 120 min at 20°C. Four different solvents were tested, i.e., H_2_O, MeOH, a mixture of MeOH : H_2_O (50 : 50, *v*/*v*), and a mixture of EtOH : H_2_O (30 : 70, *v*/*v*). After extraction, the sample was filtered with a filter paper and lyophilized with a LyovaporTM L-200 (Buchi, Cornaredo, Italy). The lyophilized SCG extracts were kept in darkness at -20°C until use. Before high-performance liquid chromatography tandem mass spectrometry (HPLC-MS/MS) analysis, 5 mL of MeOH (1 mg mL^−1^) was added to the lyophilized extract (5 mg), and the mixture was sonicated for 10 min and filtered with a 0.2 *μ*m pore size filter.

### 2.3. HPLC-MS/MS Triple Quadrupole

HPLC-MS/MS studies were performed following a previous procedure [35]. Briefly, the system was composed of an Agilent 1290 Infinity series and a Triple Quadrupole 6420 from Agilent Technology (Santa Clara, CA) equipped with an electrospray ionization (ESI) source operating in the negative and positive ionization modes. The separation of 30 analytes was achieved on a Kinetex PFP analytical column (100 × 2.1 mm, particle size 2.6 *μ*m) from Phenomenex (Torrance, CA, USA). The mobile phase was obtained mixing (W) water and (M) methanol, both with 0.1% of formic acid. The elution was carried out in gradient mode (flow rate of 0.2 mL min^−1^). The composition of the mobile phase varied as follows: 0–2 min, isocratic condition, 20% M; 2–15 min, 80% M; 15–18 min, isocratic condition, 80% M; 18–23 min, 100% M; and 23–35 min, 20% M. The injection volume was 2 *μ*L, and the column was set at 30°C. The drying gas in the ionization source was at 350°C. The flow rate of the gas was 10 L min^−1^, the nebulizer pressure was 25 psi, and the capillary voltage was 4000 V. The dynamic “multiple reaction monitoring” (dynamic MRM) mode was used for detection, and the quantification was realized by integrating the dynamic MRM peak areas. The most abundant product ion was used for quantitation, and the other to confirm the analyte. In Table 1, the selected ion transitions and the mass spectrometer parameters comprising the definite time window for each compound (*Δ* retention time) are listed.

### 2.4. Total Phenolic and Flavonoid Contents and DPPH Radical Scavenging Activity

The total phenolic content (TPC) was measured spectrophotometrically according to the method developed by Siatka and Kašparová [36] with some modifications. In particular, 0.5 mL of extract solution (1 mg mL^−1^ in methanol), 2.5 mL of Folin–Denis reagent solution, and 7 mL of Na_2_CO_3_ (7.5% *w*/*w* in water) solution were added to the test tubes. The reaction mixture was maintained at 25°C for 2 h in the dark, and the absorption was measured at 765 nm. Gallic acid was used as a reference compound, and the TPC in the extracts was calculated using gallic acid calibration curve and expressed as mg of gallic acid equivalents (GAE) per g of dry weight of SCG extract.

The total flavonoid content (TFC) of each extract was evaluated as reported in [37] with some modification. 0.5 mL of extract solution (1 mg mL^−1^), 0.15 mL of NaNO_2_ (0.5 M), 3.2 mL of methanol (30% *v*/*v*), and 0.15 mL of AlCl_3_·6H_2_O (0.3 M) were added in a 15 mL test tube. 5 min later, 1 mL of NaOH (1 M) was added and the solution was mixed well before measuring the absorbance at 506 nm. Rutin (0 to 100 mg L^−1^) was used to make the standard calibration curve for TFC following the procedure described above. TFC was reported as mg of rutin equivalents (RE) per g of dried extract.

The in vitro antioxidant activity of the extracts was measured as ability to scavenge the radical 2,2-diphenyl-1-picrydrazyl (DPPH) as reported in [38] with some modifications. Briefly, 0.5 mL of extract solution (1 mg mL^−1^ in methanol) was added to 4.5 mL of ethanolic solution of DPPH (0.1 mM) in a 15 mL test tube and allowed to stand for 30 min in the dark at 25°C. The DPPH reduction was evaluated spectrophotometrically at 517 nm. The % of DPPH scavenging was obtained following the formula: %*I* = [(*A*_control_ − *A*_sample_)/*A*_control_] × 100. *A*_control_ and *A*_sample_ indicate the absorbance obtained in the absence and presence of antioxidants, respectively. The scavenging activity of the extracts was reported as the IC_50_ value (*μ*g mL^−1^), the extract concentration which causes a 50% DPPH inhibition. The IC_50_ value was calculated by interpolation from the linear regression analysis. Trolox® (1–50 *μ*g mL^−1^) was considered as a reference antioxidant.

### 2.5. Cell Cultures and Treatments

The SH-SY5Y cell line was purchased from Sigma-Aldrich (ECACC 94030304) (St. Louis, MO, USA) and was grown in high-glucose DMEM supplemented with 10% (*v*/*v*) of FBS, 2 mM L-glutamine, 50 U/mL of penicillin, and 50 *μ*g/mL of streptomycin, as previously reported [39]. Cells were used for experiments after inducing their differentiation with all-trans retinoic acid (10 *μ*M) for 7 days.

Differentiated SH-SY5Y were treated with different concentrations of the SCG extracts for 24 h and then exposed to 700 *μ*M H_2_O_2_ for 1.0 h in 1% FBS DMEM.

BV-2 murine microglial cells were a kind gift of Prof. Elisabetta Blasi (University of Modena and Reggio Emilia, Modena, Italy). Cells were cultured in high-glucose DMEM supplemented with 10% (*v*/*v*) of low-endotoxin FBS (Euroclone, Milano), 2 mM L-glutamine, 50 U/mL of penicillin, and 50 *μ*g/mL of streptomycin. The cells were maintained in a humidified incubator at 37°C with 5% CO_2_ and subcultured using Trysin-EDTA.

BV-2 cells were pretreated with the SCG extracts at different concentrations for 24 h before the addition of 100 ng mL^−1^ LPS for 24 h.

### 2.6. Cell Viability Assay

Cell viability was evaluated by measuring MTT reduction as previously reported [40]. Briefly, at the end of each experiment, the cell medium was removed from 96-well tissue culture plates, and the cells were incubated with 0.5 mg mL^−1^ of MTT solution. The incubation time was 30 min for BV-2 cells and 90 min for SH-SY5Y cells. After removing the MTT solution, DMSO was added to lyse the cells. The presence of formazan was evaluated spectrophotometrically at 570nm using a microplate spectrophotometer (VICTOR3 V Multilabel Counter; PerkinElmer, Wellesley, MA, USA). Data are reported as percentage with respect to controls. Control cells are considered as 100% cell viability.

### 2.7. Trypan Blue Assay

SH-SY5Y were differentiated and treated with the extracts (50 *μ*g mL^−1^) and after 24 h cells were stained with 0.4% trypan blue. The viability was evaluated in Countess™ Cell Counting Chamber Slides (Invitrogen, Carlsbad, CA, USA) using the Countess® Automated Cell Counter (Invitrogen). The number of the cells was determined for each sample, and dead cells were discriminated by the incorporation of trypan blue. Percent of viability was calculated as follows:

Viability (%) = ( Live cell number/Total cell number) × 100.

### 2.8. DCFH-DA Assay

Intracellular ROS levels were evaluated using the fluorescent DCFH-DA probe as previously reported [41]. Briefly, at the end of each experiment, 10 *μ*M DCFH-DA solution in DMEM 1% FBS without phenol red was added to the cells allowed to stand for 30 min. PBS was added after removing DCFH-DA solution. Cell fluorescence was measured using 485 nm excitation and 535 nm emission on a microplate spectrofluorometer (VICTOR3 V Multilabel Counter, PerkinElmer).

### 2.9. RNA Extraction

RNeasy Mini Kit (QIAGEN GmbH, Hilden, Germany) was used to extract total RNA. The quality and quantity of RNA were evaluated by a NanoVue Spectrophotometer (GE Healthcare, Milano, Italy).

### 2.10. Real-Time Polymerase Chain Reaction (PCR)

Reverse-transcription of 1 *μ*g of the extracted RNA to cDNA was performed using iScript cDNA Synthesis Kit (Bio-Rad, Hercules, CA, USA), according to the supplier's instructions. To perform PCR, 2.5 *μ*L (12.5 ng) of cDNA, 5 *μ*L SsoAdvanced Universal SYBR Green Supermix (Bio-Rad), and 0.5 *μ*L (500 nM) of each primer were added to a PCR tube. In Tables 2 and 3, the primers used (Sigma-Aldrich, Milan, Italy) are listed. Two different reference genes were used: GAPDH rRNA for microglial cells and RPS18 for neuronal cells. cDNA amplification was started at 95°C for 30 s to activate the polymerase, followed by 40 cycles of 5 s at 95°C and 30 s at 60°C. Normalized expression levels were calculated relative to control cells according to the 2^−*ΔΔ*CT^ method.

### 2.11. Western Immunoblotting

Cells were washed with ice-cold PBS and lysed on ice using 50 mM Tris, 0.1% Triton X-100, 150 mM NaCl, and 2 mM EGTA/EDTA containing mammalian protease inhibitor mixture (1 : 100 dilution), 1 mM sodium pyrophosphate, 10 mg/mL phenylmethylsulfonyl fluoride, 1 mM sodium vanadate, and 50 mM sodium fluoride. Samples were boiled for 5 min before separation on 4-20% SDS-polyacrylamide gels (20 *μ*g/lane). A nitrocellulose membrane was used to transfer proteins (Hybond-C; GE Healthcare, Buckinghamshire, UK) in Tris-glycine buffer at 110 V for 90 min. The membranes were incubated in blocking buffer prepared with 5% (*w*/*v*) bovine serum albumin (BSA) and then incubated with anti-HO1 (Cell Signaling Technology, Beverly, MA) (1 : 1000 dilution) and anti-*β*-actin (Sigma Aldrich–Merck) (1 : 5000 dilution) as internal loading control, overnight at 4°C on a three-dimensional rocking table. Targeted proteins were visualized using ClarityTM Western ECL Substrate (Bio-Rad). Densitometric analysis of specific immunolabeled bands was performed using ImageJ software.

### 2.12. Flow Cytometry

To evaluate the surface expression of TLR4 receptor on BV-2 cells, 1 × 10^5^ cells were seeded in 12-well tissue culture plates. At the end of each experiment, cells were washed with PBS and detached with accutase solution. The cells were centrifuged at 300 g for 5 min. The cell pellet was washed twice by centrifugation and resuspension in washing buffer (0.2% BSA-PBS), in 1.5 mL tubes. After removing the supernatant, the cells were resuspended with FITC-conjugated rabbit anti-TLR4 antibody (Stressmarq, cat. no. SPC-200), 1 : 100 dilution in 0.2% BSA-PBS, then incubated for 30 min in the dark at 37°C according to the manufacturer's instructions. After antibody incubation, the cells were washed twice as above. After supernatant aspiration, the samples were appropriately diluted to 5 × 10^5^ cells mL^−1^ and finally resuspended in BSA 0.1% PBS for flow cytometry reading. Guava® easyCyte™ 5 HT instrument was used to collect all raw data. FlowJo software was used to analyze the mean fluorescence intensity (MFI). Unstained samples were used as negative controls.

### 2.13. Immunofluorescence Confocal Microscopy

BV-2 cells were cultured directly on glass coverslips in 6-well plates. Cells were then fixed with 2% paraformaldehyde in PBS for 15 min at room temperature and permeabilized with Triton 0.1% for 10 min, after which they were treated with a polyclonal antibody (1 : 500) against NF-*κ*B p65 overnight. Following extensive washing with PBS, cells were incubated with a secondary Alexa Fluor 488-conjugated anti-rabbit IgG antibody diluted 1 : 1000 in PBS for 1 h at room temperature. Nuclei were stained with 1 *μ*g mL^−1^ of 4′-6-diamidino-2-phenylindole (DAPI). Slides were analyzed with a C2 Plus confocal laser scanning microscope (Nikon Instruments, Firenze, Italy). Images were processed using NIS Element Imaging Software (Nikon Instruments, Firenze, Italy).

### 2.14. Statistical Analysis

The experiments were carried out at least in triplicate, and values were reported as mean ± standard error. The differences among groups were evaluated by one-way ANOVA followed by Dunnett's or Bonferroni's test (Prism 5; GraphPad Software, San Diego, CA) (for cell culture data). Differences at the level *p* < 0.05 were considered statistically significant.

## 3. Results and Discussion

### 3.1. Bioactive Compounds in Different SCG Extracts

Before extract analysis, the analytical method has been validated testing linearity, limit of detection (LOD), limit of quantification (LOQ), and repeatability. The calibration curves were plotted on seven points by injecting seven different concentrations of mixtures of 30 analytes, and the respective determination coefficients (*R*^2^) were calculated. The *R*^2^ for each monitored molecule was ≥0.9937, which implied good linearity. LOD and LOQ were evaluated by injecting gradually lower concentration of standard mixtures, and the concentration with signal-to-noise ratio (SNR) of 3 was assigned to LOD that with SNR of 10 was assigned to LOQ. The LODs obtained ranged from 0.3 to 50 *μ*g L^−1^, while the LOQs were between 1 and 200 *μ*g L^−1^. The repeatability has been tested by injecting five replicates of three different concentrations of the standard solutions on the same day (run-to-run precision) and on three consecutive days (day-to-day precision). Relative standard deviation (RSD) % was utilized to define the intraday repeatability or run-to-run precision and interday repeatability or day-to-day precision. Run-to-run precision was between 1.7% and 3.9%, whereas day-to-day precision was between 4.3% and 7.4%.

Four SCG extracts were prepared, i.e., MeOH (E1), H_2_O (E2), MeOH : H_2_O (50 : 50, *v*/*v*) (E3), and EtOH : H_2_O (70 : 30, *v*/*v*) (E4). The content of bioactive compounds (*μ*g g^−1^ of dry weight extract) measured in each SCG extract is listed in Table 4. All extracts were prepared using ultrasound-assisted extraction (UAE), and the analytes were quantified using an HPLC-MS/MS system. The higher content of bioactive compounds was found in EtOH : H_2_O extract (71629.19 ± 3025.85 *μ*g g^−1^) followed by MeOH : H_2_O (69891.35 ± 3102.12 *μ*g g^−1^), MeOH (58796.31 ± 2756.32 *μ*g g^−1^), and H_2_O (56792.60 ± 2521.98 *μ*g g^−1^). Therefore, the solvent type significantly influenced the analyte extraction, and the EtOH : H_2_O and MeOH : H_2_O were shown to be the most efficient. Similar outcomes were reported in another recent work [21] which dealt with the chemical composition and some biological properties of different SCG and coffee silverskin (CS) extracts. Caffeine (41047.71-52346.41 ± 1896.25-2536.98 *μ*g g^−1^) and 5-*O*-caffeoylquinic acid (5-CQA) (7569.25-13256.35 ± 305.21-499.74 *μ*g g^−1^) were the most abundant in all extracts followed by chlorogenic acids, i.e., 3-*O*-caffeoylquinic acid (3-CQA) (2324.33-4317.31 ± 100.89-185.42 *μ*g g^−1^) and 3,5-*O*-dicaffeoylquinic acid (3,5-diCQA) (902.34-1325.98 ± 58.12-88.23 *μ*g g^−1^). Andrade et al. [42] have reported similar levels of caffeine in SCG, using UAE with different solvents, finding the best results with dichloromethane (38200.00 *μ*g g^−1^) and ethanol (25700.00 *μ*g g^−1^). Considering that the use of dichloromethane should be discouraged since it is associated with both acute and chronic toxicity in humans, including respiratory, central nervous system, and cardiovascular toxicity, carcinogenicity, and genotoxicity [43], the use of ethanol was shown to be a good choice according to the extraction efficiency and at the same time the environmental impact. As reported in Table 1, all seven unconjugated phenolic acids were recovered in all extracts and the most abundant were caffeic (81.58-220.71 ± 1.65-10.36 *μ*g g^−1^), ferulic (82.47-155.32 ± 3.45-5.89 *μ*g g^−1^), and vanillic acid (65.23-122.36 ± 2.36-5.14 *μ*g g^−1^). Such molecules were also the most abundant in different SCG extracts prepared using a filter coffeemaker preceded by a defatting process with petroleum ether, as reported by Monente et al. [44]. At slightly lower concentrations, we identified gallic acid (57.62-112.29 ± 2.65-4.26 *μ*g g^−1^) and shikimic acid (23.11-86.70 ± 1.23-3.26 *μ*g g^−1^), an important metabolite involved in the biosynthesis of aromatic amino acids L-phenylalanine, L-tyrosine, and L-tryptophan in microorganisms and plants (shikimate pathway) [45, 46]. Among the eleven flavonoids monitored in the current study (kampferol 3-glucoside, quercetin, quercitrin, hyperoside, rutin, (+)-catechin, (-)-epicatechin, cyanidin 3-glucoside, delphinidin 3,5-diglucoside, naringin, and resveratrol), nine of them have been found in the SCG extracts. The most abundant was (-)-epicatechin, a flavonoid of flavan-3-ol subclass, which occurred only in MeOH (87.23 ± 2.98 *μ*g g^−1^) and MeOH : H_2_O (85.11 ± 2.22 *μ*g g^−1^) extracts and two molecules of flavonol subclass, i.e., quercetin (3.15-3.96 ± 0.15-0.13 *μ*g g^−1^) and its glycoside rutin (3.33-10.11 ± 0.15-0.61 *μ*g g^−1^). Interestingly, cyanidin 3-glucoside, an anthocyanin that occurs in coffee skin and pulp [47], has been found in all extracts ranging from 1.02 to 2.03 ± 0.05-0.09 *μ*g g^−1^ but not delphinidin 3,5-diglucoside. As already reported by Angeloni et al. (2020), iridoids and secoiridoids did not occur in spent coffee and probably in coffee beans. On the other hand, an alkaloid first isolated from the *Cinchona* tree known as a potent antimalarial agent, namely, quinine (1.44-3.23 ± 0.07-0.12 *μ*g g^−1^) [48], and a xanthone of Gentian plant [49], namely, isogentisin (1.12-1.65 ± 0.04-0.06 *μ*g g^−1^), were detected in all SCG extracts.

### 3.2. Total Phenolic and Flavonoid Contents and DPPH Radical Scavenging Activity of SCG Extracts

Table 5 reports the content of the phenolic and flavonoid compounds and the radical scavenging activity of different SCG extracts. The TPC has been spectrophotometrically measured, and data are reported as mg of gallic acid equivalents (GAE) per g of dry weight of SCG extract. The highest levels of phenolic compounds were found in E4 (112.65 ± 4.53 mg GAE/g) followed by E3 (95.12 ± 3.56 mg GAE/g), E1 (88.75 ± 2.13 mg GAE/g), and E2 (69.32 ± 2.11 mg GAE/g) extract. These levels were higher than those reported by other works when a simply solid-liquid extraction was employed [50–52]. For instance, Bravo et al. found SCG extracts with TPC of 17.44 ± 0.26 mg GAE/g using water for analyte extraction [52]. On the other hand, Al-Dhabi et al., who performed UAE at different conditions, obtained higher levels of TPC (32.81-36.23 mg GAE/g) [53]. The use of ultrasound during the extraction process increases the mass transfer due to acoustic cavitation effect generated by ultrasonic waves [54], and this can be the reason together with the coffee variability of higher TPC obtained in the current research. The total content of chlorogenic acids, one of the most important class of phenolic compounds in coffee, measured by the HPLC system, was characterized by the same abovementioned ranking, i.e., EtOH : H_2_O (18512.04 ± 895.32 *μ*g g^−1^) followed by MeOH : H_2_O (17869.34 ± 925.26 *μ*g g^−1^), MeOH (17252.40 ± 823.12 *μ*g g^−1^), and H_2_O (10795.92 ± 772.65 *μ*g g^−1^). In contrast, the highest level of TFC, expressed as mg of rutin equivalents (RE) per g of dried extract, was obtained in MeOH : H_2_O (6.29 ± 0.23 mg RE/g) followed by MeOH (6.17 ± 0.16 mg RE/g), EtOH : H_2_O (5.56 ± 0.12 mg RE/g) and H_2_O (3.15 ± 0.14 mg RE/g) extract. These data are consistent with HPLC-MS/MS studies on the total content of monitored flavonoids since they can be ranked in the following order MeOH : H_2_O (107.53 ± 7.25 *μ*g g^−1^) > MeOH (100.92 ± 5.98 *μ*g g^−1^) > EtOH : H_2_O (25.92 ± 1.08 *μ*g g^−1^) > H_2_O (14.01 ± 0.65 *μ*g g^−1^). The radical scavenging activity of SCG extracts has been evaluated by DPPH assay, and it was expressed as the IC_50_ value (*μ*g mL^−1^) which is the concentration of the extract necessary to cause 50% of DPPH inhibition. The solvent type influenced the antioxidant capacity of the extracts, and the highest radical scavenging activities were obtained with EtOH : H_2_O (196.25 ± 6.87 *μ*g mL^−1^) and MeOH (215.35 ± 7.42 *μ*g mL^−1^). Notably, the H_2_O extract (585.32 ± 25.32 *μ*g mL^−1^) was the worst in terms of antioxidant capacity and it was characterized by lower content of bioactive compounds as well. The latter together with an inefficient extraction of low-polar compounds could be the reason of lower antioxidant activity. In fact, some lipophilic compounds which usually occur in coffee, e.g., diterpenes and tocopherols, are known as powerful antioxidants [55, 56].

### 3.3. Neuroprotective Activity of SCG Extracts

#### 3.3.1. Antioxidant Activity

**Figure 1:**
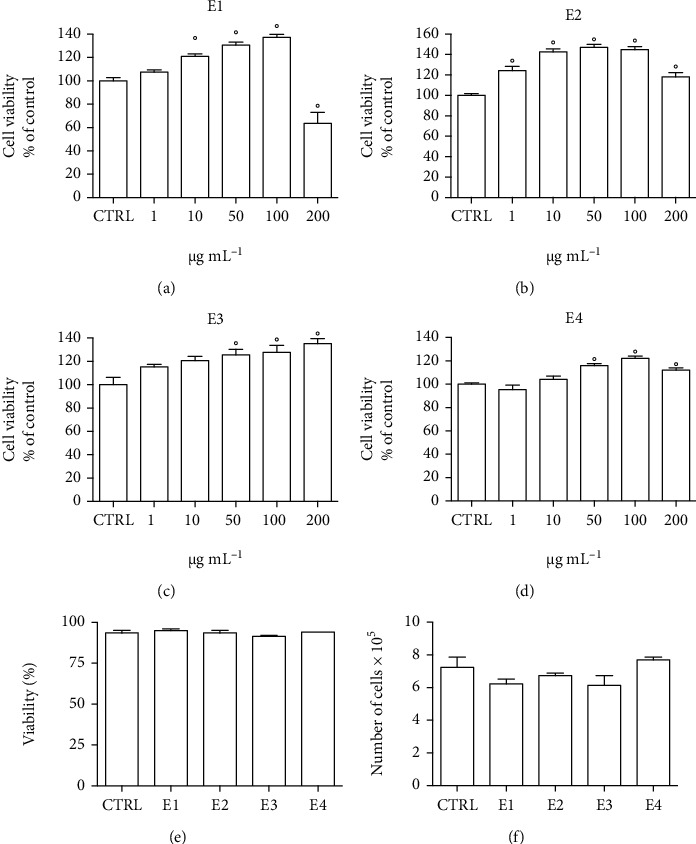
Effect of the different extracts on viability of SH-SY5Y cells. (a–d) Cells were treated with 1–200 *μ*g mL^−^1 of each extract for 24 h, and cell viability was evaluated by MTT assay. (e, f) Cells were treated with 50 *μ*g mL^−1^ of each extract for 24 h, and the trypan blue assay was used to measure cell number and cell viability. Each bar represents means ± SEM of at least four independent experiments. Data were analyzed by one-way ANOVA followed by Dunnett's test. °*p* < 0.05 with respect to CTRL.

The in vitro antioxidant activity of E1, E2, E3, and E4 has been investigated in neuron-like SH-SY5Y cells differentiated with retinoic acid. To study the potential cytotoxicity of E1, E2, E3, and E4, cells were treated with 1–200 *μ*g mL^−1^ of the four extracts for 24 h and MTT assay was used to measure cell viability (Figures 1(a)–1(d)). The extracts were not cytotoxic up to 200 *μ*g mL^−1^ except the E1 extract that induced a significant reduction of cell viability at 200 *μ*g mL^−1^. Interestingly, the treatment with the extracts led to a significant increase of cell viability. As MTT evaluates cell viability as the enzymatic conversion of the tetrazolium compound to water-insoluble formazan crystals by dehydrogenases occurring in the mitochondria of living cells [57], we can suppose that this cell viability increase could be caused by an intensification of mitochondrial respiration. All extracts are rich in caffeine that has been associated to an increased mitochondrial content due to the upregulation of peroxisome proliferator-activated receptor gamma coactivator 1-alpha (PGC-1*α*) that modulates the nuclear respiratory factors 1 and 2 (NRF1/2) and mitochondrial transcription factor A (TFAM) [58–61]. Moreover, the treatment with caffeine of isolated human muscle fibers showed a direct effect on the mitochondrial activity by increasing the respiration rate and concomitantly decreasing the mitochondrial membrane potential [62]. In a previous study, we observed a significant increase of cell viability of differentiated SH-SY5Y cells treated with extracts containing caffeine, and these new data reinforce the hypothesis that caffeine could be the compound responsible for this effect [5]. Of course, further investigations are needed to determine a direct involvement of caffeine in the enhancement of mitochondrial respiration in SH-SY5Y cells. To clarify if the observed increase in cell viability is only linked to an increase in mitochondrial activity, we measured cell viability with a different viability assay. Differentiated SH-SY5Y cells were treated with 50 *μ*g mL^−1^ of each extract for 24 h, and cell viability was measured by the trypan blue assay (Figure 1(e)) that is based on the principle that living cell membranes are intact and exclude trypan blue, whereas the dead cells are permeable to the dye. Of note, all treatments did not increase cell viability suggesting that the increase in cell viability measured by MTT assay is related to an increase in mitochondrial activity. Another hypothesis to explain the observed increase in cell viability could be a corresponding increase in cell proliferation. To investigate this aspect, differentiated SH-SY5Y cells were treated with 50 *μ*g mL^−1^ of each extract for 24 and the total cell number was counted (Figure 1(f)). Interestingly, the treatments did not modify the cell number, confirming that the observed increase in cell viability measured by MTT assay is related to a higher rate of mitochondrial respiration and not to an increased proliferation.

**Figure 2:**
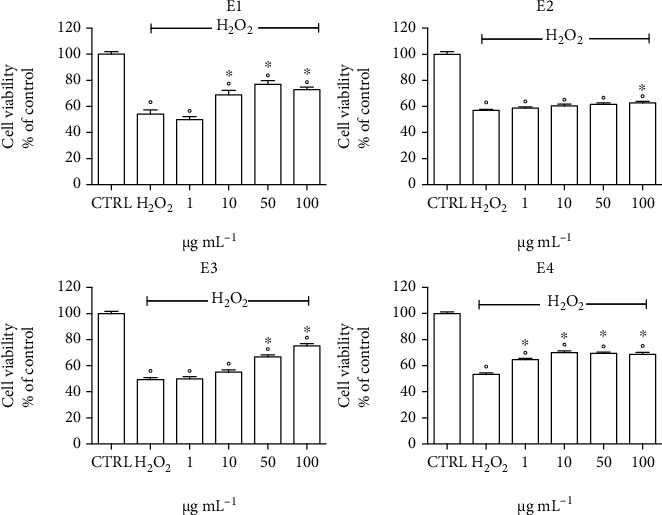
Cytoprotective activity of the extracts in SH-SY5Y cells exposed to H_2_O_2_. Cells were pretreated with 1–100 *μ*g mL^−1^ of each extract for 24 h, exposed to 700 *μ*M H_2_O_2_ for 1 h before measuring cell viability by MTT assay. Each bar represents means ± SEM of at least four independent experiments. Data were analyzed by one-way ANOVA followed by Bonferroni's test. °*p* < 0.05 with respect to CTRL; ^∗^*p* < 0.05 with respect to H_2_O_2_.

The antioxidant activity of the extracts has been evaluated pretreating SH-SY5Y cells with 1-100 *μ*g mL^−1^ of the extracts for 24 h before exposing the cells to 700 *μ*M H_2_O_2_ to induce oxidative stress (Figure 2). At the lowest concentrations, only the E4 extract significantly increased cell viability with respect to H_2_O_2_-treated cells, meanwhile, at 10 *μ*g mL^−1^, E1 was also able to significantly increase cell viability. E3 significantly counteracted oxidative stress at 50 *μ*g mL^−1^ and E2 only at the highest tested concentrations. Of note, at 50 *μ*g mL^−1^, E1 increased cell viability with respect to peroxide-treated cells by about 22%, meanwhile E3 and E4 by about 16%, evidencing a higher ability of E1 in counteracting oxidative stress-induced damage in SH-SY5Y cells.

**Figure 3:**
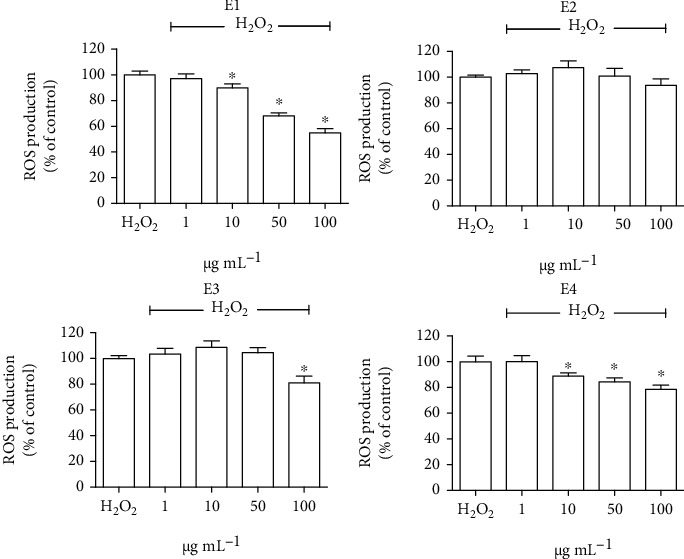
ROS levels of SH-SY5Y cells treated with the extracts and exposed to H_2_O_2_. Cells were pretreated with 1–100 *μ*g mL^−1^ of the different extracts for 24 h and then treated with H_2_O_2_. The peroxide-sensitive probe DCFH-DA was used to measure ROS production. Data are expressed as percentage with respect to H_2_O_2_-treated cells. Each bar represents means ± SEM of at least four independent experiments. Data were analyzed by one-way ANOVA followed by Dunnett's test. ^∗^*p* < 0.05 with respect to H_2_O_2_.

To further investigate the antioxidant activity of the extracts, SH-SY5Y cells were treated with 1-100 *μ*g mL^−1^ of each extract and the DCFH-DA assay was used to evaluate the effect on intracellular ROS production (Figure 3). The results showed that E1 and E4 were the most effective ones in reducing ROS levels, meanwhile E3 reduced ROS levels only at the 100 *μ*g mL^−1^ and E2 did not influence this parameter. These results confirm that E1 and E4 are the extracts with the strongest antioxidant activity.

These biological results on the antioxidant activity of the extracts are in agreement with the results obtained by DPPH assay (Table 2). In particular, in SH-SY5Y cells, E1 and E4 extracts were the most effective ones in terms of antioxidant activity, meanwhile E2 showed the lowest activity. As previously underlined, the low antioxidant capacity of E2 could be caused by an inefficient extraction of low-polar compounds, which usually occur in coffee and are known for their elevated antioxidant activity [55, 56].

**Figure 4:**
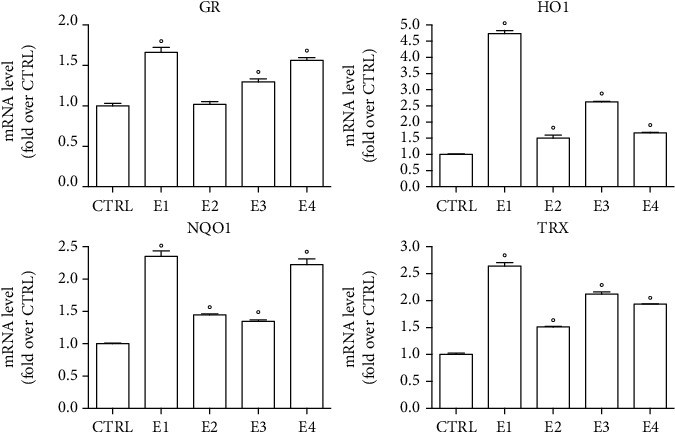
Effect of the extracts on the mRNA level of antioxidant enzymes in SH-SY5Y cells. Cells were treated with 50 *μ*g mL^−1^ of each extract for 5 h. GR, HO1, NQO1, and TRX mRNA levels were determined by RT-PCR. Data are reported as relative abundance in respect to control cells (CTRL). Each bar represents mean ± SEM of three independent experiments. Data were analyzed with a one-way ANOVA followed by the Dunnett's test. °*p* < 0.05 vs. CTRL.

Evidence is cumulating which shows that many phytochemicals exert antioxidant activity through an indirect antioxidant mechanism, i.e., enhancing the expression of antioxidant enzymes and cytoprotective proteins [63–66]. To verify if the extracts modulate the endogenous antioxidant system, we treated SH-SY5Y cells with 50 *μ*g mL^−1^ of each extract before analyzing the expression of the antioxidant enzymes glutathione peroxidase (GR), heme oxygenase 1 (HO1), NADP(H) oxidoreductase 1 (NQO1) and thioredoxin reductase by RT-PCR (Figure 4). All the extracts significantly upregulated HO1, NQO1, and TRX, meanwhile GR expression was significantly increased only by E1, E3, and E4.

**Figure 5:**
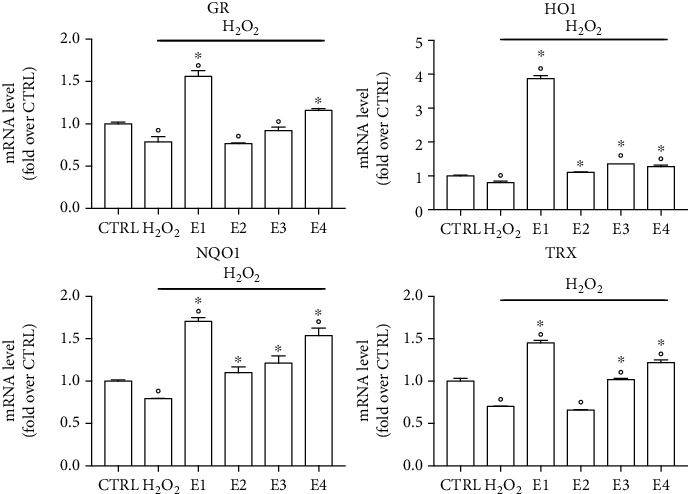
Effect of the extracts on the mRNA level of SH-SY5Y in the presence of H_2_O_2_. Cells were pretreated with 50 *μ*g mL^−1^ of each extract and after 5 h exposed to H_2_O_2_ 700 *μ*M for 1 h. GR, HO1, NQO1, and TRX mRNA levels were determined by RT-PCR. Data are reported as relative abundance in respect to control cells (CTRL). Each bar represents mean ± SEM of three independent experiments. Data were analyzed with a one-way ANOVA followed by the Bonferroni's test. °*p* < 0.05 vs. CTRL; ^∗^*p* < 0.05 vs. H_2_O_2_.

We also evaluated the expression of these antioxidant enzymes in the presence of H_2_O_2_. In particular, SH-SY5Y cells were pretreated with 50 *μ*g mL^−1^ of each extract and then exposed to H_2_O_2_ before analyzing mRNA levels of GR, HO1, NQO1, and TRX (Figure 5). H_2_O_2_ exposure significantly reduced the expression of all the tested genes in respect to control cells. Considering the short H_2_O_2_ exposure, the observed downregulation of these genes could be probably ascribed to the H_2_O_2_-induced oxidation of the corresponding mRNA. E1 was able to significantly upregulate all the four genes with respect to both H_2_O_2_ and controls. E2 treatment did not influence GR and TRX expressions with respect to H_2_O_2_-treated cells, meanwhile slightly but significantly upregulated HO1 and NQO1 expressions. E3 significantly increased mRNA levels of HO1, NQO1, and TRX with respect to H_2_O_2_-treated cells and upregulated HO1 and TRX with respect to control cells. E4 significantly increased the expression of HO1, NQO1, and TRX with respect to both H_2_O_2_ and controls, meanwhile significantly upregulated GR only with respect to H_2_O_2._

**Figure 6:**
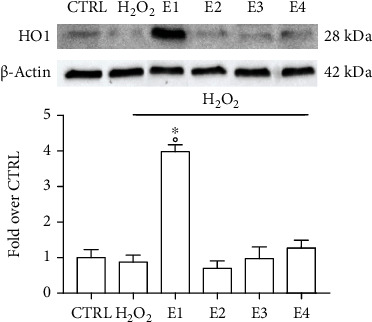
Protein level of HO1 in SH-SY5Y cells treated with the extracts and exposed to H_2_O_2_. Cells were treated with E1, E2, E3, and E4 (50 *μ*g mL^−1^) and after 24 h exposed to H_2_O_2_ 700 *μ*M for 1 h. Immunoblotting was performed using anti-HO1. Data are expressed as fold over CTRL and normalized by *β*-actin. Each bar represents mean ± SEM of three independent experiments. Data were analyzed with a one-way ANOVA followed by the Bonferroni's test. °*p* < 0.05 vs. CTRL; ^∗^*p* < 0.05 vs. H_2_O_2_.

Considering the strong upregulation of HO1 with respect to the other tested genes, we performed an immunoblotting analysis to confirm HO1 induction also at a protein level. SH-SY5Y cells were pretreated with 50 *μ*g mL^−1^ of each extract and then exposed to H_2_O_2_ before western blot analysis (Figure 6). H_2_O_2_ exposure reduced HO1 protein levels with respect to control cells, even if not significantly. On the contrary, E1 strongly and significantly increased the expression of HO1, confirming the expression data.

Interestingly, E1, with respect to the other extracts, showed a marked ability to upregulate the four antioxidant enzymes both in the absence and in the presence of H_2_O_2_ suggesting that the higher ability of E1 to protect SH-SY5Y cells against oxidative stress could be ascribed to its ability to strongly upregulate the endogenous antioxidant system.

Considering the characterization of the extracts in terms of bioactive compound content (Table 1), E1 showed the highest content of (-)-epicatechin and isogentisin with respect to the other extracts. Of note, no correlation was evidenced between (-)-epicatechin content and the different parameters tested to analyze the antioxidant activity of the extracts. On the other hand, isogentisin content was positively correlated with the protection against H_2_O_2_ (*r* = 0.9745, *p* < 0.05) and GR expression (*r* = 0.9575, *p* < 0.05) and inversely correlated with ROS levels (*r* = −0.9604, *p* < 0.05). The xanthone isogentisin is a characteristic constituent found in plants such as Gentianaceae [67]. Very few studies investigated its biological activity. In particular, isogentisin has been shown to counteract smoking-caused injury in human umbilical vein endothelial cells (HUVECs) [68] and to inhibit monoamine oxidase types A and B in rat brains [69, 70]. Monoamine oxidase inhibitors are considered important agents for the treatment of depression, anxiety, and neurodegenerative disorders, including Alzheimer's and Parkinson's diseases [71, 72]. From this point of view, further studies will be necessary both to investigate the antioxidant activity of pure isogentisin in neuron cells and to verify if the E1 extract also can act as a monoamine oxidase inhibitor.

### 3.3.2. Anti-Inflammatory Activity

The in vitro anti-inflammatory activity of the extracts has been investigated in microglial BV-2 cells. Microglia are equivalent to macrophages in the brain and represent the first and most important line of defense in the central nervous system. Under physiological conditions, microglia have a key role in neuronal survival through the production of neurotrophic factors and the phagocytosis of dead cells, cellular debris, protein aggregates, and invading pathogens [73]. However, excessively activated microglia can lead to neurotoxicity through the production of proinflammatory mediators such as tumor necrosis factor alpha (TNF-*α*), nitric oxide, interleukin-1*β* (IL-1*β*), IL-6, and ROS [74]. Different studies have shown that microglia play an important role in the onset and progression of neurodegenerative diseases such as Parkinson's disease and Alzheimer's disease [75–77].

**Figure 7:**
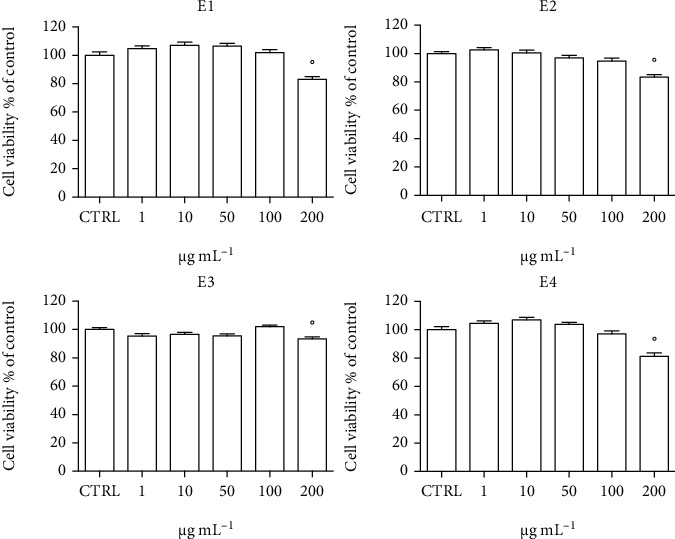
Effect of the different extracts on cell viability of BV-2 cells. Cells were treated with 1–200 *μ*g mL^−1^ of each extract for 24 h, and MTT assay was used to obtain cell viability. Each bar represents means ± SEM of at least four independent experiments. Data were analyzed by one-way ANOVA followed by Dunnett's test. °*p* < 0.05 with respect to CTRL.

Prior to investigating the effect of the extracts on BV-2 microglia-mediated neuroinflammation, we assessed the potential cytotoxicity of E1, E2, E3, and E4 on BV-2 microglial cells using MTT assay (Figure 7). The extracts were not cytotoxic up to 100 *μ*g mL^−1^, meanwhile all extracts were cytotoxic at 200 *μ*g mL^−1^ as demonstrated by a significant reduction of cell viability at this concentration with respect to control cells.

**Figure 8:**
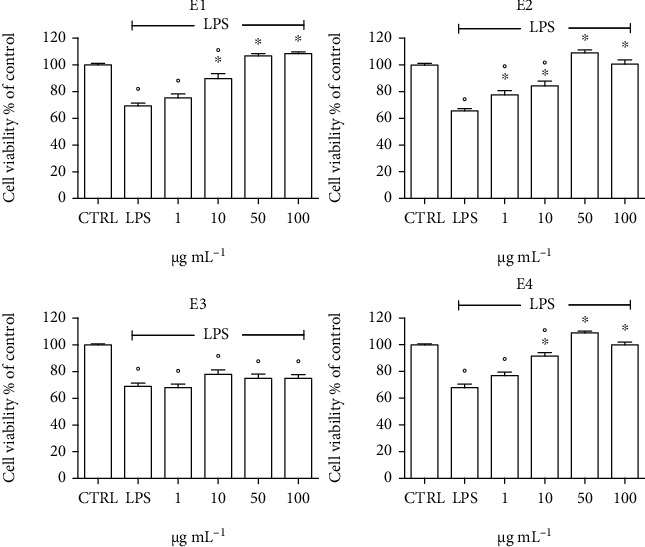
Cytoprotective activity of the extracts in BV-2 cells activated by LPS. Cells were pretreated with 1–100 *μ*g mL^−1^ of each extract for 24 h, activated with 100 ng mL^−1^ LPS for 24 h, and MTT assay was used to measure cell viability. Each bar represents means ± SEM of at least four independent experiments. Data were analyzed by one-way ANOVA followed by Bonferroni's test. °*p* < 0.05 with respect to CTRL; ^∗^*p* < 0.05 with respect to LPS.

The anti-inflammatory activity of the extracts was evaluated pretreating BV-2 cells with different concentrations (1-100 *μ*g mL^−1^) of the extracts for 24 h before exposing the cells to lipopolysaccharide (LPS) to induce inflammation (Figure 8). LPS is the most widely used inflammatory mediator to activate microglial cells in vitro and triggers the proinflammatory signaling cascade [78, 79]. LPS treatment significantly reduced cell viability with respect to control cells by ~40%. Interestingly, E1, E2, and E4 were able to significantly increase cell viability with respect to LPS-treated cells, and at 50 *μ*g mL^−1^, all of them were able to maintain cell viability to a value comparable to control cells. On the other hand, E3 did not show any protective effect against LPS-induced damage.

**Figure 9:**
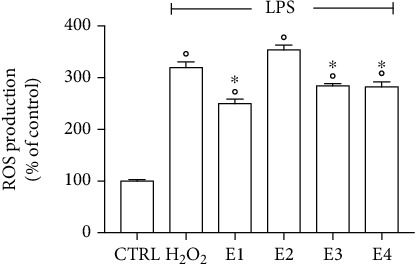
ROS levels of BV-2 cells treated with the extracts and activated by LPS. Cells were pretreated with 50 *μ*g mL^−1^ of the different extracts for 24 h and then activated by LPS. The peroxide-sensitive probe DCFH-DA was used to measure ROS production. Data are expressed as % compared to control cells (CTRL). Each bar represents means ± SEM of at least four independent experiments. Data were analyzed by one-way ANOVA followed by Bonferroni's test. °*p* < 0.05 with respect to CTRL; ^∗^*p* < 0.05 with respect to H_2_O_2_.

As it has been shown that LPS induces oxidative stress [80, 81], we measured intracellular ROS levels in BV-2 cells pretreated with 50 *μ*g mL^−1^ of the extracts for 24 h and then activated by LPS (Figure 9). As expected, LPS significantly increased intracellular ROS levels with respect to controls. E1, E3, and E4 significantly reduced ROS levels compared to LPS-treated cells, meanwhile E2 did not modify ROS levels with respect to LPS-treated cells, in agreement with the results obtained in SH-SY5Y cells.

**Figure 10:**
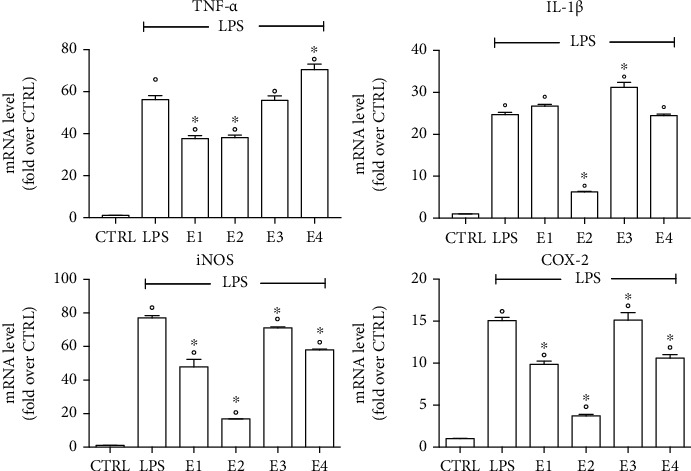
Expression of proinflammatory cytokines and enzymes in BV-2 cells treated with the extracts. Cells were treated with E1, E2, E3, and E4 (50 *μ*g mL^−1^) for 24 h, exposed to 100 ng mL-1 LPS for 24 h and TNF-*α*, IL1-*β*, iNOS, and COX-2 mRNA levels were measured by RT-PCR. Data are expressed as relative abundance compared to untreated cells. Each bar represents the mean ± SEM of three independent experiments. Data were analyzed with a one-way ANOVA followed by Bonferroni's test. °*p* < 0.05 vs. CTRL; ^∗^*p* < 0.05 vs. LPS.

Since proinflammatory cytokines and enzymes including tumor necrosis factor *α* (TNF-*α*), interleukin 1*β* (IL-1*β*), cyclooxygenase 2 (COX-2), and inducible nitric oxide synthase (iNOS) are crucial mediators of neuroinflammation, we next measured the effects of the extracts on these inflammatory mediators in LPS-stimulated BV-2 microglial cells (Figure 10). BV-2 microglial cells were pretreated with 50 *μ*g mL^−1^ of E1, E2, E3, and E4, followed by LPS for 24 h. Total RNA was isolated, and proinflammatory cytokine and enzyme expressions were measured using RT-PCR. As expected, LPS significantly increased the expression of TNF-*α*, IL-1*β*, COX-2, and iNOS with respect to control cells. In agreement with the MTT data, E3 did not show any ability to inhibit LPS-induced expression of TNF-*α* and COX-2 and significantly increased the expression of IL-1*β* with respect to LPS. Moreover, E3 was able to significantly reduce iNOS expression with respect to LPS, but the extent of this reduction was very small, maintaining iNOS expression to levels strongly higher than those measured in control cells. E1 and E4 had no effect on LPS-induced IL-1*β* expression, meanwhile they were able to significantly reduce iNOS and COX-2 expression with respect to LPS-treated cells. These two extracts showed opposite behaviors with respect to TNF-*α* expression: E1 significantly inhibited LPS-induced expression of this cytokine, on the contrary E4 significantly increased its expression with respect to LPS-treated cells. Of note, E2 was the most effective extract and significantly and strongly inhibited the expression of all proinflammatory mediators analyzed. These results are partially in agreement with the data on cell viability that showed that E1, E2, and E4 had a similar effect in counteracting LPS-induced damage, and all of them were able to completely protect cells against neuroinflammation at 50 *μ*g mL^−1^. This discrepancy could be explained by the different mechanisms by which these extracts counteract LPS-induced damage. The mechanism behind E2 protection is easy to understand as this extract exerts a strong anti-inflammatory activity that significantly and strongly reduces the expression of all proinflammatory mediators investigated. On the contrary, the protective activity of E1 and E4 cannot be explained only in terms of their ability in reducing proinflammatory cytokine and enzyme expression levels. Taking into consideration the results also obtained in SH-SY5Y, we can suggest that E1 and E4 were able to protect BV-2 cells against LPS-induced damaged thanks to their antioxidant activity. In fact, it is widely accepted that LPS generates ROS that trigger oxidative stress and cell damage [82–84]. Of note, E1, E3 and E4, but not E2 at 50 *μ*g mL^−1^, significantly reduced ROS levels in BV-2 cells.

**Figure 11:**
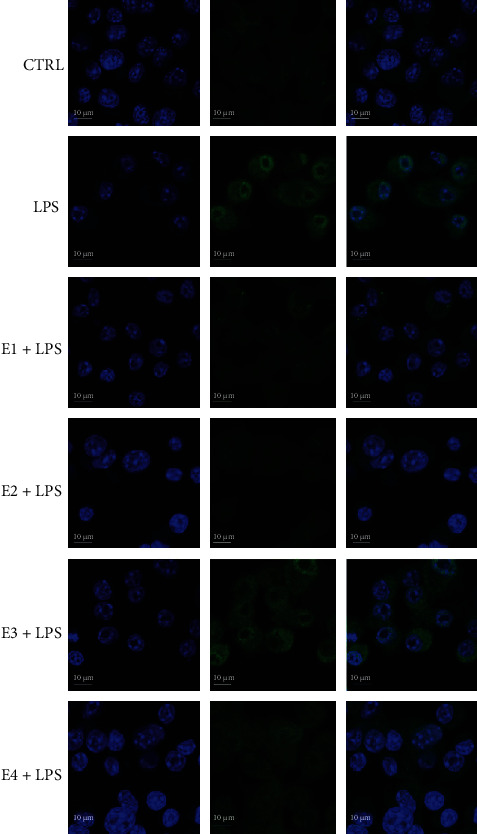
NF-*κ*B nuclear translocation in BV-2 cells treated with the extracts. Cells were treated with 50 *μ*g mL^−1^ of each extracts for 24 h, activated by LPS for 24 h. Cells were immunostained with a primary antibody against NF-*κ*B p65, followed by secondary Alexa Fluor 488-conjugated anti-rabbit IgG antibody (green), and cell nuclei (blue) were visualized with DAPI. Scale bar: 10 *μ*m. Tests were performed in triplicate.

The NF-*κ*B pathway is a key mediator of inflammation and is activated via Toll-like receptors (TLRs) resulting in increased cytokine and chemokine production [85]. It has been observed that the activation of NF-*κ*B and release of its subunits play a crucial role in the onset and progression of neurodegenerative disorders [86, 87]. Moreover, transcription of TNF-*α*, IL-1*β*, iNOS, and COX-2 is regulated by the transcription factor NF-*κ*B. To further elucidate the mechanisms of the extracts on the inhibition of the expression of these proinflammatory mediators in BV-2 cells, the effect of E1, E2, E3, and E4 on NF-*κ*B activation was investigated by confocal microscopy (Figure 11). BV-2 cells were pretreated with 50 *μ*g mL^−1^ of the extracts, exposed to LPS for 24 h and immunostained with a primary antibody against NF-*κ*B p65, followed by Alexa Fluor 488-conjugated secondary antibody. LPS induced a strong increase in NF-*κ*B protein levels and triggered its translocation to the nucleus with respect to control cells. In agreement with the previous data, E1, E2, and E4 reduce NF-*κ*B protein levels with respect to LPS-treated cells, confirming their anti-inflammatory ability. Of note, E1 and E2 maintained NF-*κ*B protein levels to values comparable to control cells. On the other hand, E3 maintained NF-*κ*B protein levels to a value comparable to LPS-treated cells.

**Figure 12:**
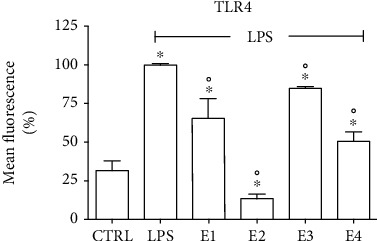
Effect of the extracts on cell surface expression of TLR4 in BV-2 cells. Cells were treated with E1, 50 *μ*g mL^−1^ of each extract for 24 h, exposed to 100 ng mL^−1^ LPS for 24 h, and TLR4 surface expression was evaluated by flow cytometry. Data are expressed as percentage compared to LPS-activated cells. Each bar represents the mean ± SEM of three independent experiments. Data were analyzed with one-way ANOVA followed by Bonferroni's test. °*p* < 0.05 vs. CTRL, ^∗^*p* < 0.05 vs. LPS.

One of the main receptors mediating the activation of microglia and release of proinflammatory mediators is Toll-like receptor 4 (TLR4). LPS is a well-characterized ligand of TLR4 [88]. Dimerization of TLR4 induces the downstream activation of NF-*κ*B signaling, triggering the activation of immune cells such as microglia [89]. On these bases, we further investigated the effects of the extracts on TLR4 cell surface expression by cytofluorimetric analysis (Figure 12). LPS induced a strong and significant increase of TLR4 surface expression with respect to control cells. According to the previous results, E2 showed the strongest effect in significantly reducing TLR4 surface expression both with respect to LPS-treated cells and control cells. E1, E3, and E4 significantly reduced TLR4 surface expression with respect to LPS-treated cells and, in agreement with the previous results, E3 was the least effective.

Considering the characterization of the extracts in terms of bioactive compound content (Table 2), it is not possible to find any correlation among the anti-inflammatory activity and the presence of specific compounds in the extracts. E2, the most effective extract in counteracting neuroinflammation, does not contain compounds that are not present in the other extracts; moreover, all characterized compounds are present at a lower concentration with respect to the other extracts. Therefore, we can hypothesize that E2 could contain some bioactive compounds that we have not identified. Further researches are needed to better characterize the composition of this extract.

## 4. Conclusions

The extract analysis evidenced that the different solvents had a profound impact on the composition of the extracts. In particular, the higher content of potential bioactive compounds was found in EtOH : H_2_O and MeOH : H_2_O extracts. Interestingly, the biological data revealed that the richest extracts in terms of compounds were not the most effective in counteracting oxidative stress and inflammation. The MeOH extract showed the strongest antioxidant activity in neuron-like SH-SY5Y cells, reducing intracellular ROS levels and upregulating endogenous antioxidant enzymes such as NQO1, GR, TRX, and HO1. This effect seems to be related to its higher content of isogentisin with respect to the other extracts. The H_2_O extract elicited the highest anti-inflammatory activity, markedly reducing the expression of proinflammatory mediators by the TLR4/NF-*κ*B pathway. Of note, none of the identified compounds in the H_2_O extract can explain its higher anti-inflammatory activity with respect to the other extracts. For this reason, further studies should be carried out to better characterize this extract and identify potential bioactive compounds responsible for its anti-inflammatory activity. In conclusion, the antioxidant and anti-inflammatory properties of the extracts suggest that SCGs are a valuable source of nutraceuticals that could be used to prevent/counteract neurodegeneration.

## Figures and Tables

**Table 1:** HPLC-MS/MS acquisition parameters, working as a dynamic “multiple reaction monitoring” mode, including retention time (Rt) and delta retention time (*Δ* Rt) for each transition.

No.	Compounds	Precursor ion (m/z)	Product ion (m/z)	Fragmentor (V)	Collision energy (V)	Polarity	Retention time (Rt) (min)	Delta retention time (*Δ* Rt)
1	Shikimic acid	173	173	87	0	Negative	1.40	3
			—	—	—	—		
2	Gallic acid	169	125^a^	92	12	Negative	2.37	3
			51		36			
3	Loganic acid	375	213^a^	126	8	Negative	3.13	3
			113		16			
4	3-Caffeoylquinic acid	353	191^a^	102	12	Negative	3.58	3
			179		12			
5	Swertiamarin	419	179^a^	100	4	Negative	4.89	3
			89		16			
6	Gentiopicroside	357	177^a^	50	10	Positive	5.33	3
			73		28			
7	(+)-Catechin	289	245^a^	121	8	Negative	5.48	3
			109		24			
8	Delphinidin-3,5-diglucoside	463	300^a^	165	24	Negative	5.64	3
			271		48			
9	Sweroside	403	125^a^	102	12	Negative	5.95	3
			179		4			
10	5-Caffeoylquinic acid	353	191^a^	92	12	Negative	6.22	3
			85		48			
11	Caffeine	195	138^a^	107	20	Positive	6.50	3
			110		24			
12	Cyanidin-3-glucoside	449	287^a^	121	20	Positive	6.50	3
			403		16			
13	Vanillic acid	167	108^a^	78	16	Negative	6.70	3
			152		8			
14	Caffeic acid	179	135^a^	87	12	Negative	6.87	3
			134		24			
15	(-)-Epicatechin	289	245^a^	126	8	Negative	7.03	3
			109		20			
16	Syringic acid	197	182^a^	92	8	Negative	7.48	3
			123		20			
17	p-Coumaric acid	163	119^a^	83	12	Negative	8.47	3
			93		32			
18	Ferulic acid	193	134^a^	88	12	Negative	9.16	3
			178		8			
19	3,5-Dicaffeoylquinic acid	515	353^a^	117	8	Negative	9.82	3
			191		28			
20	Quinine	325	79^a^	135	44	Positive	10.1	5
			81		32			
21	Naringin	579	271^a^	210	32	Negative	10.17	3
			151		48			
22	Rutin	609	300^a^	195	40	Negative	10.34	3
			271		50			
23	Hyperoside	463	300^a^	160	24	Negative	10.43	3
			271		44			
24	*trans*-Cinnamic acid	149	131^a^	44	8	Positive	10.79	3
			77		36			
25	Resveratrol	227	185^a^	131	12	Negative	10.92	3
			143		20			
26	Amarogentin	585	227^a^	145	16	Negative	11.05	3
			245		16			
27	Kaempferol-3-glucoside	447	284^a^	163	24	Negative	11.24	3
			227		50			
28	Quercitrin	447	300^a^	155	24	Negative	11.24	3
			301		16			
29	Quercetin	301	151^a^	126	16	Negative	13.03	3
			179		12			
30	Isogentisin	257	242^a^	116	16	Negative	16.31	3
			214		24			

^a^These product ions were used for quantification; the others to confirm the analytes.

**Table 2:** List of primers for real-time PCR in SH-SY5Y cells.

Gene	Primer
RPS18 forward	5′CAGAAGGATGTAAAGGATGG3 ′
RPS18 reverse	5′TATTTCTTCTTGGACACACC3 ′
GR forward	5′GACCTATTCAACGAGCTTTAC3 ′
GR reverse	5′CAACCACCTTTTCTTCCTTG3 ′
NQO1 forward	5′AGTATCCACAATAGCTGACG3 ′
NQO1 reverse	5′TTTGTGGGTCTGTAGAAATG3 ′
HO1 forward	5′CAACAAAGTGCAAGATTCTG3 ′
HO1 reverse	5′TGCATTCACATGGCATAAAG3 ′
TRX forward	5′AGACAGTTAAGCATGATTGG3 ′
TRX reverse	5′AATTGCCCATAAGCATTCTC3 ′

**Table 3:** List of primers for real-time PCR in BV-2 cells.

Gene	Primer
GAPDH forward	5′ACCACAGTCCATGCCATCAC3 ′
GAPDH reverse	5′TCCACCACCCTGTTGCTGTA3 ′
IL-1*β* forward	5′GTTCCCATTAGACAACTGCACTACAG3 ′
IL-1*β* reverse	5′GTCGTTGCTTGGTTCTCCTTGTA3 ′
TNF-*α* forward	5′CCCCAAAGGGATGAGAAGTTC3 ′
TNF-*α* reverse	5′CCTCCACTTGGTGGTTTGCT3 ′
iNOS forward	5′CCTCCTCCACCCTACCAAGT3′
iNOS reverse	5′CACCCAAAGTGCTTCAGTCA3′
COX2 forward	5′TGGGGTGATGAGCAACTATT3 ′
COX2 reverse	5′AAGGAGCTCTGGGTCAAACT3 ′

**Table 4:** Bioactive compounds content (*μ*g g^−1^ of dry weight extract) in spent coffee ground extracts.

No.	Analytes^a^	E1 (MeOH)	E2 (H_2_O)	E3 (MeOH : H_2_O)	E4 (EtOH : H_2_O)
1	Shikimic acid	38.52 ± 1.84	23.11 ± 1.23	86.70 ± 3.26	71.15 ± 3.12
2	Gallic acid	87.65 ± 3.33	57.62 ± 2.65	112.29 ± 4.26	75.91 ± 2.81
3	Loganic acid	n.d.^c^	n.d.	n.d.	n.d.
4	3-CQA^b^	3637.65 ± 157.21	2324.33 ± 100.89	3587.15 ± 163.24	4317.31 ± 185.42
5	Swertiamarin	n.d.	n.d.	n.d.	n.d.
6	Gentiopicroside	n.d.	n.d.	n.d.	n.d.
7	(+)-Catechin	0.95 ± 0.04	n.d.	1.25 ± 0.05	1.02 ± 0.04
8	Del 3,5-diglu^b^	n.d.	n.d.	n.d.	n.d.
9	Sweroside	n.d.	n.d.	n.d.	n.d.
10	5-CQA^b^	12699.32 ± 483.26	7569.25 ± 305.21	13256.35 ± 499.74	12868.75 ± 401.68
11	Caffeine	41047.71 ± 1896.25	45568.32 ± 2121.56	51236.74 ± 2036.15	52346.41 ± 2536.98
12	Cya 3-glu^b^	1.56 ± 0.07	1.02 ± 0.05	1.85 ± 0.08	2.03 ± 0.09
13	Vanillic acid	65.23 ± 2.36	82.65 ± 3.33	122.36 ± 5.14	105.41 ± 4.21
14	Caffeic acid	81.58 ± 1.65	103.28 ± 4.78	170.83 ± 5.98	220.71 ± 10.36
15	(-)-Epicatechin	87.23 ± 2.98	n.d.	85.11 ± 2.22	n.d.
16	Syringic acid	23.56 ± 1.01	44.15 ± 1.87	43.65 ± 2.10	78.63 ± 3.88
17	*p*-Coumaric acid	8.36 ± 0.32	9.45 ± 0.29	15.23 ± 1.12	28.12 ± 1.15
18	Ferulic acid	82.47 ± 3.45	87.54 ± 2.65	118.96 ± 4.13	155.32 ± 5.89
19	3,5-diCQA^b^	915.43 ± 55.32	902.34 ± 58.12	1025.84 ± 64.32	1325.98 ± 88.23
20	Quinine	1.44 ± 0.07	1.69 ± 0.06	2.75 ± 0.10	3.23 ± 0.12
21	Naringin	n.d.	0.62 ± 0.03	0.40 ± 0.02	0.47 ± 0.02
22	Rutin	3.33 ± 0.15	5.36 ± 0.33	8.75 ± 0.52	10.11 ± 0.61
23	Hyperoside	0.98 ± 0.04	0.86 ± 0.03	0.75 ± 0.03	1.23 ± 0.06
24	*trans*-Cin acid^b^	6.27 ± 0.24	5.44 ± 0.30	6.49 ± 0.32	8.11 ± 0.35
25	Resveratrol	n.d.	n.d.	n.d.	n.d.
26	Amarogentin	n.d.	n.d.	n.d.	n.d.
27	Kae 3-glu^b^	1.54 ± 0.06	1.03 ± 0.05	1.97 ± 0.08	2.84 ± 0.11
28	Quercitrin	0.47 ± 0.02	0.28 ± 0.01	0.74 ± 0.03	1.12 ± 0.05
29	Quercetin	3.42 ± 0.12	3.15 ± 0.13	3.96 ± 0.15	3.87 ± 0.11
30	Isogentisin	1.65 ± 0.06	1.12 ± 0.04	1.23 ± 0.05	1.45 ± 0.05

Total compounds		58796.31 ± 2756.32	56792.60 ± 2521.98	69891.35 ± 3102.12	71629.19 ± 3025.85

^a^Each sample was analyzed in triplicate (*n* = 3); ^b^3-CQA: 3-caffeoylquinic acid; 3,5-diCQA: 3,5-dicaffeoylquinic acid; 5-CQA: 5-caffeoylquinic acid; Del 3,5-diglu: delphinidin 3,5-diglucosiede; Cya 3-glu: cyanidin 3-glucoside; *trans*-Cin acid: *trans*-cinnamic acid; Kae 3-glu: kaempferol 3-glucoside; ^c^n.d.: not detectable.

**Table 5:** Total phenolic content (TPC), total flavonoid content (TFC), and DPPH radical scavenging activity of the different spent coffee ground extracts.

Extracts	Total phenolic content (mg GAE/g)	Total flavonoid content (mg RE/g)	DPPH IC50 (*μ*g/mL)
E1 (MeOH)	88.75 ± 2.13	6.17 ± 0.16	215.35 ± 7.42
E2 (H_2_O)	69.32 ± 2.11	3.15 ± 0.14	585.32 ± 25.32
E3 (MeOH : H_2_O)	95.12 ± 3.56	6.29 ± 0.23	298.44 ± 13.12
E4 (EtOH : H_2_O)	112.65 ± 4.53	5.56 ± 0.12	196.25 ± 6.87

## Data Availability

The data used to support the findings of this study are available from the corresponding author upon request.
